# A Salvage Approach for Iatrogenic Iliac Vein Injury During Kidney Transplantation

**DOI:** 10.3390/jcm14092917

**Published:** 2025-04-23

**Authors:** Pāvils Plūme, Igors Losevs, Viktors Ševeļovs, Olga Jegorova, Aleksandrs Maļcevs, Vadims Suhorukovs, Maija Radziņa, Jānis Jušinskis

**Affiliations:** 1 Latvian Transplant Center, Pauls Stradiņš Clinical University Hospital, LV-1002 Riga, Latvia; 2 Department of Surgery, Riga Stradiņš University, LV-1007 Riga, Latvia; 3 Institute of Diagnostic Radiology, Pauls Stradiņš Clinical University Hospital, LV-1002 Riga, Latvia; 4 Faculty of Medicine and Life Sciences, University of Latvia, LV-1050 Riga, Latvia; 5 Radiology Department, Riga Stradiņš University, LV-1007 Riga, Latvia

**Keywords:** kidney transplant, iliac vein injury, vascular injury, case report

## Abstract

**Background and objectives**: Iatrogenic injury to the external iliac vein is a rare surgical complication during kidney transplantation. It can compromise the use of the vein for anastomosis and adversely affect venous return from the ipsilateral lower extremity. **Case presentation**: We present an innovative salvage technique for addressing iatrogenic injuries to the external iliac vein occurring during its dissection from the surrounding tissues. This approach involves the attachment of an allograft renal vein to the distal segment of the divided external iliac vein using end-to-end anastomosis, while the proximal segment is anastomosed to the allograft renal vein using an end-to-side technique. Early postoperative ultrasound evaluations indicated sufficient venous return from both the transplanted kidney and the lower extremity. A recent follow-up, 12 years post-transplantation, confirmed sustained vascularization and venous return from the allograft. **Conclusions**: The described technique provides an effective solution for managing significant external iliac vein injuries during kidney transplant procedures. It facilitates the salvage of the vein for anastomosis with the allograft in the ipsilateral iliac fossa without the need for vascular tissue replacement or altering the anastomosis site.

## 1. Introduction

Kidney transplantation is widely recognized as the preferred treatment for patients with end-stage kidney disease, providing superior overall survival rates and enhanced quality of life compared to dialysis [1]. In the traditional heterotopic kidney transplant procedure, the donor renal artery and vein are typically anastomosed to the recipient’s external iliac vessels. However, intraoperative challenges such as damage or thrombosis of the external iliac vein can complicate the procedure and have traditionally been grounds for canceling the transplant [2]. While the existing literature has been widely addressing iliac vein thrombosis, there is limited discussion on iatrogenic damage to the iliac vessels, with reports focusing on arterial damage and mostly post-operative vascular complications [3]. Here, we report a rare case of iatrogenic injury to the right external iliac vein during kidney transplant surgery, utilizing a functional salvage approach. This technique involved anastomosing the allograft renal vein to the distal segment of the external iliac vein in an end-to-end fashion, while the proximal portion of the external iliac vein was anastomosed to the allograft renal vein in an end-to-side manner.

## 2. Case Presentation

### 2.1. Patient Perspective

The patient was a 51-year-old female with an end-stage renal disease due to focal segmental glomerulosclerosis (FSGS) diagnosed 14 years prior. Hemodialysis was initiated in 2010 with a left lower arm arteriovenous fistula (AVF). Her medical history was also significant for arterial hypertension, dyslipidemia, and obesity (BMI: 36 kg/m^2^). The patient was placed on the kidney transplant waiting list on July 2011. In May 2012, a matching left kidney from a deceased brain-dead donor (DBD) was transplanted on the right side. Pre-transplant ultrasound showed classical iliac vessel anatomy.

### 2.2. Surgical Procedure

The donor kidney was prepared on the back table using ice and Custodiol HTK tissue preservation fluid. The allograft showed classic anatomy, featuring one artery with a Carrel patch, one vein, and one ureter.

**Figure 1:**
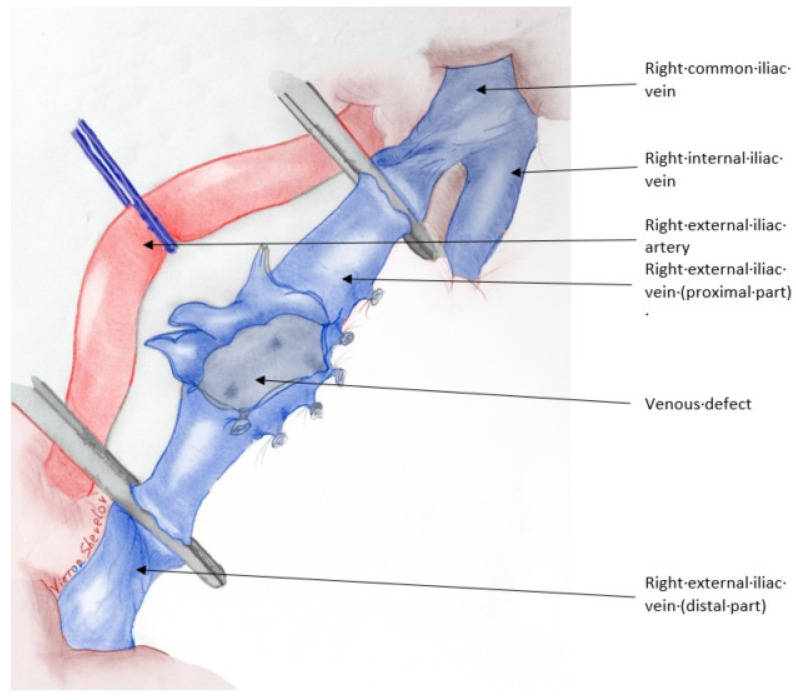
The damaged right external iliac vein after clamping.

**Figure 2:**
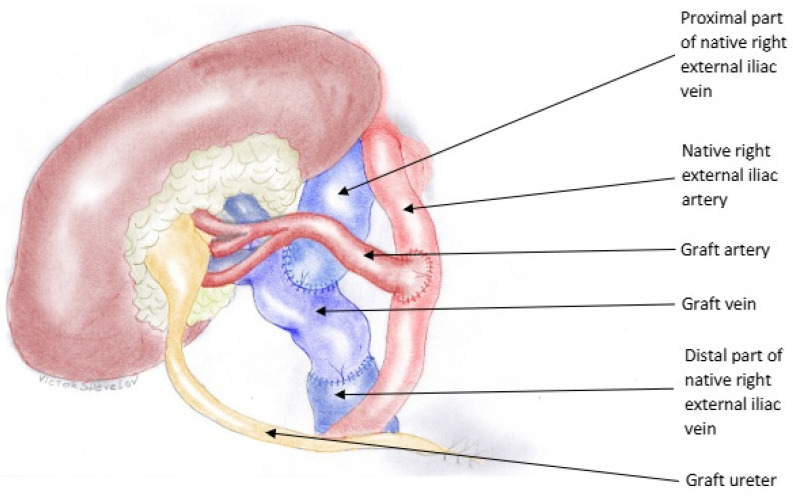
The reconstructed venous anatomy. The renal allograft has been attached to the distal external iliac vein in an end-to-end fashion. The proximal external iliac vein has been attached to the allograft renal vein in an end-to-side fashion.

The recipient was positioned supine, and a modified Gibson incision was made to access the extraperitoneal right iliac fossa. The intraperitoneal organs were retracted laterally, and a Codman–Bookwalter retractor system was used for optimal visualization. The right external iliac artery and vein were dissected free from the surrounding tissues. The dissection of the right external iliac vein was complicated by the presence of multiple minor side branches on the lateral and posterior aspects of the vessel. During mobilization, a branch on the lateral surface of the iliac vein was inadvertently damaged (Figure 1). An attempt to repair this injury led to an extension of the laceration, rendering the vein unsuitable for conventional end-to-side anastomosis. A novel salvage technique was employed, in which the renal vein was anastomosed to the distal external iliac vein in an end-to-end fashion. Subsequently, the proximal portion of the external iliac vein was anastomosed to the renal vein in an end-to-side configuration (Figure 2). The anastomoses were completed with a continuous 6-0 Prolene suture. The arterial reconstruction was then performed using an aortic patch in an end-to-side manner, according to the standard technique at our center. The total anastomotic time was 30 min, cold ischemia time—23 h. Immediate urine output was not observed. Subsequently a stented anti-reflux ureteroneocystostomy was performed. Early ultrasound imaging indicated preserved vascularization, normal renal resistive index (RI), and optimal venous outflow from the graft. No perirenal hematomas were noted.

Induction immunosuppression was achieved utilizing basiliximab and methylprednisolone followed by a triple-drug regimen: cyclosporin, mycophenolate mofetil, and prednisolone for maintenance. An unfractionated heparin infusion at a rate of 400 IU per hour was administered for the first two postoperative days. This was followed by a standard anticoagulation protocol using low-molecular-weight heparin (LMWH) at a dosage of 3000 IU twice daily for two weeks.

**Figure 3:**
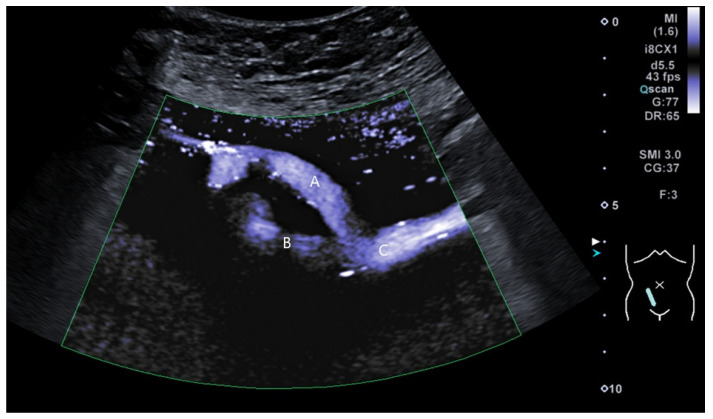
The allograft renal vein is shown anastomosed to the distal part of the external iliac vein. A—allograft renal vein; B—proximal external iliac vein; C—distal external iliac vein. Ultrasound superb vascular imaging mode (SMI) mode.

**Figure 4:**
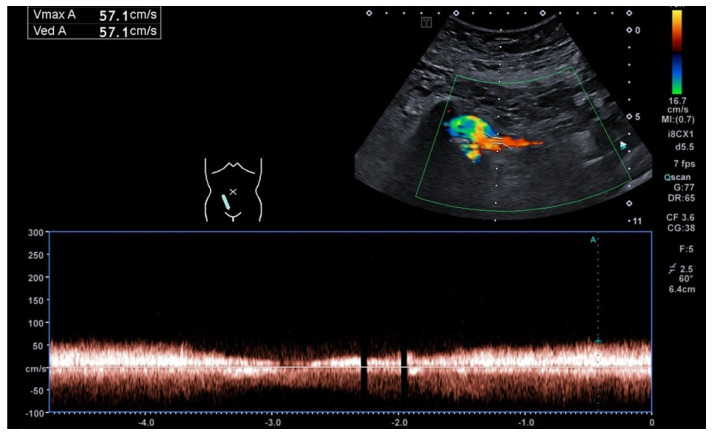
Pulse Wave Doppler (PWD) of the renal transplant vein graft shows continuous flow up to 57 cm/s.

The early postoperative period was complicated by Grade IA rejection and delayed graft function, which were managed with three doses of Solumedrol. The patient was discharged on postoperative day 24 following successful management of the rejection episode. During the follow-up period, the patient developed ureteric stenosis in the transplanted allograft, which was corrected in 2017. The most recent follow-up in November 2024 revealed that the patient had preserved kidney function with chronic kidney disease (CKD) Stage IIIB. Contrast Enhanced Ultrasound (CEUS) revealed preserved vascularization and optimal venous outflow from the allograft (Figure 3 and Figure 4).

## 3. Discussion

### 3.1. Vascular Complications in Renal Transplants

The current literature indicates that the most common vascular complications during and after renal transplants include transplant renal artery stenosis (TRAS), renal graft thrombosis, arteriovenous fistulas (AVFs), flow-limiting dissections, and pseudo-aneurysms [3]. While most vascular complications in kidney transplantation are associated with arterial issues, the primary complication concerning the venous system is renal vein thrombosis, which typically develops in the early postoperative period and is one of the leading causes of early graft loss within the first month after transplantation [4]. Another major issue regarding the iliac venous system is iliac vein thrombosis before transplantation and there are multiple articles discussing potential strategies to manage this issue. In cases of bilateral iliac vein thrombosis, the optimal practice involves anastomosing the renal vein to the inferior vena cava [5]. The gonadal veins and portal system veins such as inferior mesenteric vein and splenic vein can also serve as suitable options for allograft venous drainage, provided they are adequately sized to ensure sufficient drainage [6,7]. There is a scarcity of studies specifically addressing the management of iatrogenic vascular injuries during the transplant procedure, with most research focusing on kidney or iliac artery dissections [8,9].

### 3.2. Management of Iliac Vein Injuries

While there is limited literature on iliac vein injuries specifically during a kidney transplant, extensive information exists on managing isolated iliac vessel injuries. Options include primary repair, vessel ligation, the use of synthetic or biological patches, or prosthetic bypass formation [10]. Primary repair with sutures is only recommended for minor injuries and is associated with narrowing the venous lumen, leading to stenosis. Another option for major iliac vein injury, especially in the case of uncontrollable bleeding, is vessel ligation. Although it is associated with higher mortality rates, it does not increase the risk of compartment syndrome, lower extremity amputation, deep vein thrombosis (DVT), or pulmonary embolism (PE) [11]. Ligation of the right external iliac vein in our case would prevent the use of the left donor kidney for transplantation in the already prepared right iliac fossa, prompting us to pursue a salvage approach. A case report by H. Demitras et al. in the *Journal of Updates in Cardiovascular Medicine* describes using a synthetic conduit or, where viable, a saphenous vein graft to replace the damaged iliac vein segments [12]. Another report in the *Texas Heart Institute Journal* by Del Campo C. et al. highlights the supremacy of autologous grafts or xenografts in venous repair over synthetic grafts. In their study, the approach involved using a custom-made bovine pericardium tubular graft to replace an injured common iliac vein segment during lumbar spine surgery [13]. Kayle J. Krause, in *Global Surgery*, suggests using a bovine pericardial patch for venoplasty, which circumvents the risk of venous narrowing associated with primary repair [14].

### 3.3. Case Specific Considerations

In our case, significant damage to the iliac vein necessitated urgent management before proceeding with the transplant. The primary focus was to stop the bleeding and restore the disrupted venous backflow from the right leg. Additionally, we aimed to use the right iliac fossa for the transplant since the incision had already been made and exposure achieved. Therefore, we considered a viable salvage option. Primary repair of the injury was deemed unfeasible due to its size and extent. Suturing could potentially narrow the venous lumen, leading to stenosis and increasing the risk of deep vein thrombosis (DVT). The saphenous vein was considered too narrow to serve as a conduit and could only function as a patch for repair. However, using it in this manner would eliminate the site for anastomosis. At our institution, synthetic patches are not commonly used for venous repair due to their inferior outcomes compared to biological materials. Additionally, the unfamiliarity with the procedure and limitations in resources precluded the use of synthetic materials. The use of xenografts was not an option due to their unavailability at the time of surgery. The preferred approach was to use an allogenic venous graft from the donor, obtained during procurement. It is a standard practice at our institution to graft iliac vessels to elongate the right kidney vein due to its shorter length. Unfortunately, the donor material had already been fully utilized for elongating the vein of the initially transplanted right kidney. Ultimately, our approach allowed for successful graft implantation in the patent iliac vessel, maintained venous backflow from the lower extremity, and avoided the need for additional graft tissue.

## 4. Conclusions

It is essential to emphasize the importance of backup options and alternatives in the event of iatrogenic injury to any major blood vessels during kidney transplant procedures. Preparation should include securing an adequate supply of allograft iliac vein and artery grafts from the donor during procurement. Furthermore, surgeons should be familiar with alternative repair options, including the use of synthetic, allogenic, or xenograft prostheses or patches. In cases of uncontrollable bleeding or damage that is not amenable to repair, it may be necessary to consider changing the transplant site and ligating the external iliac vein as an acceptable strategy. The described method offers a viable salvage option for addressing external iliac vein damage during kidney transplantation when primary repair is not feasible. The success of this technique hinges on the compatibility between the diameters of the recipient’s iliac vessels and the size of the allograft iliac vein, as well as the expertise of the surgeon performing the procedure.

## Data Availability

The data analyzed during the current study are not publicly available due to patient privacy and confidentiality restrictions but may be made available from the corresponding author upon request, subject to ethical approvals and data protection agreements.
